# DigitalExposome: quantifying impact of urban environment on wellbeing using sensor fusion and deep learning

**DOI:** 10.1007/s43762-023-00088-9

**Published:** 2023-03-20

**Authors:** Thomas Johnson, Eiman Kanjo, Kieran Woodward

**Affiliations:** Department of Computer Science, Nottingham Trent University, Nottingham, UK

**Keywords:** Sensor-fusion, Environment, Exposome, DigitalExposome, Machine learning, Wellbeing

## Abstract

The increasing level of air pollutants (e.g. particulates, noise and gases) within the atmosphere are impacting mental wellbeing. In this paper, we define the term ‘DigitalExposome’ as a conceptual framework that takes us closer towards understanding the relationship between environment, personal characteristics, behaviour and wellbeing using multimodal mobile sensing technology. Specifically, we simultaneously collected (for the first time) multi-sensor data including urban environmental factors (e.g. air pollution including: Particulate Matter (PM1), (PM2.5), (PM10), Oxidised, Reduced, Ammonia (NH3) and Noise, People Count in the vicinity), body reaction (physiological reactions including: EDA, HR, HRV, Body Temperature, BVP and movement) and individuals’ perceived responses (e.g. self-reported valence) in urban settings. Our users followed a pre-specified urban path and collected the data using a comprehensive sensing edge device. The data is instantly fused, time-stamped and geo-tagged at the point of collection. A range of multivariate statistical analysis techniques have been applied including Principle Component Analysis, Regression and Spatial Visualisations to unravel the relationship between the variables. Results showed that Electrodermal Activity (EDA) and Heart Rate Variability (HRV) are noticeably impacted by the level of Particulate Matter in the environment. Furthermore, we adopted Convolutional Neural Network (CNN) to classify self-reported wellbeing from the multimodal dataset which achieved an f1-score of 0.76.

## Introduction

The long-term exposure to urban environment stressors such as particulate matter, gases and noise have been found to significantly impact an individual’s behaviour and psychological health (Guite et al., 2006). The World Health Organisation (*WHO*) found that 91% of people are living in places where the air quality guidelines are not met and the use of non-clean fuels and household emissions in the atmosphere are causing over 4.2 million deaths each year (Ji, 2022). In addition, those living in some locations in the UK have a higher risk of developing serious health conditions such as higher heart rate (Kanjo et al., 2018), asthma and cardio-cerebrovascular disease where a lifetime of exposure to high-levels of pollution can result in reduced life expectancy (Air & Plan, 2021).

Recent developments in urban sensing and Internet of Things (*IoT*) has created the possibility to utilise environmental and on-body sensing tools to monitor the environment and its impact on individuals (Stamatelopoulou et al., 2018). Sensor-based technologies are becoming increasingly popular due to their availability to collect data in real-time, affordability and small size (Ueberham & Schlink, 2018). These advances continue to enable more opportunities for capturing environmental signature in urban setting by providing the mechanisms to collect and analyse objective data physiological changes and behaviour markers of mental wellbeing (Woodward et al., 2019) in real-time. In addition, the major advances and recent developments within data science have created greater opportunities to understand large multimodal datasets through machine learning, deep learning and spatial visualisations (Johnson et al., 2020).

Literature on the Exposome is lacking in terms of mental wellbeing centric investigation. For example, ExpoApp have modeled the short term health impact of high air pollution (Donaire-Gonzalez et al., 2019). A ‘Project Helix’ studied the environmental impact on individuals living in urban environments considering evidence of blood pressure, asthma, allergy related illnesses (Maitre et al., 2018). It has evidence that polluted environments around us have shown increased risk of developing serious health conditions like asthma and cardio-cerebrovascular diseases (Loh et al., 2017). Early studies including our previous work is demonstrating the impact of environmental factors on mental wellbeing (Johnson & Kanjo, 2021). However, existing research on Exposome was majorly focusing on physical illness and related issues lacking mental health centric investigation (Siroux et al., 2016). It also can be challenging to fully understand and address exposome because of its diversity, volume and quality of the data produced.

In this context, we present ‘*DigitalExposome*’ as the quantification step in understanding the relationship between the environment and mental health along with the perceived environmental responses which could potentially help in designing our cities focusing on mental wellbeing in mind. The following main research question has been addressed in this paper: How can we monitor, fuse, model and understand the person-environment interaction to help determine what makes an urban environment mentally healthy”. To answer this question , a prototype product, hardware and software is designed and developed for collecting and analysing mental health data in urban environment. Table 1, depicts the multi-sensor fusion data obtained from participants.

**Table 1:** DigitalExposome dataset variables and units

Urban Environmental Attributes	Body Physiological Reactions	Individuals’ perceived responses
Particulate Matter 1.0 ($\upmu$g/m$^{3}$)	Heart Rate (BPM)	Self-reported valence in the form of Emojis
Particulate Matter 2.5 ($\upmu$g/m$^{3}$)	Heart-Rate Variability (ms)	
Particulate Matter 10 ($\upmu$g/m$^{3}$)	ElectroDermal Activity ($\upmu$S)	
Noise (dB)	Accelerometer (m/s$^{2}$)	
Reducing Gases ($\upmu$g/m$^{3}$)	Blood Volume Pulse (millivolts)	
Oxidising Gases ($\upmu$g/m$^{3}$)	Body Temperature ($^{\circ }$C)	
Ammonia ($\upmu$mol/L)		
Carbon Dioxide (ppm)		
Volatile Organic Compound (ppm)		
People count via wireless proximity detection		

The sensing kit built for this project comprises a sensing edge (Enviro-Edge) with ten (10) embedded air quality sensors. The kit links to a custom-built smart phone app (EnvBodySens2) that collects accelerometer data, Bluetooth Low Energy (BLE) signal for people count, self-report labels, Noise, Date/Time and GPS traces. On-body data was collected using E4 Empatica. The data is instantly fused, time-stamped and geo-tagged at the point of collection. By collecting the data “in the wild” and out of the lab, paves the way for more realistic approach that can generalise to urban real-life environment. To the best of our knowledge this study developed a next generation sensing hardware prototype for unique combinatorial data collection and analysis including a comprehensive list of on-body, contextual and environmental sensors along with the user responses which has not been attempted before. Major contributions of the paper are listed as follows:

1. A real-world study with participants was organised for data collection. The correlation between environmental and physiological variables indicate that particulate matter and certain gases can be related to a decrease in heart-rate variability (HRV) and Electrodermal Activity (EDA).
2. A range of multivariate statistical analysis techniques have been applied including Principle Component Analysis, Regression and spatial visualisations (including heat maps and geometrical tessellation) to explore correlated patterns in the data and unravel the association between the attributes which might suggest a causal relationship.
3. Visualizations of the spatiality of wellbeing on three different levels, including: (i) Individual-the wellbeing of one individual in same environment - (temporal), (ii)Accumulated-the wellbeing of one individual in many environments (spatial), and (iii)Collective wellbeing- the wellbeing of group of individuals in many environments.
4. Predictive models applied to the heterogeneous multivariate attributes including, K-Nearest Neighbor, Decision Trees and Support Vector Machines, and Deep neural networks-based techniques such as Convolutional Neural Network (CNN) to extract features from the multimodal data feed which then fed in to the machine learning algorithms. The performance of the on-body modality and environment modality is compared to infer affect quality (mental wellbeing) from the data.

## Related work

Repeated and continuous human exposure to the environment and high-concentrated air pollutants have been found to increase the risk of developing serious conditions such as respiratory and cardiovascular diseases or even death (Laville, 2020). Research recently has began focusing towards how the environment can impact physical health but it also is necessary to explore how the environment can impact mental wellbeing. Pollution within the urban environment is a continual problem contributing to rising health and mental wellbeing challenges. The ability to monitor air pollutants, physiology and mental wellbeing will help unravel the relationship between the variables.

ExpoApp used a sensor fusion approach (environmental and on-body) to model the short term health impact of high air pollution. Their analysis showed those who didn’t have access to green spaces inhaled a higher rate of air pollution. A similar study monitored the environmental impact to an individual, indicating a positive correlation between the environment, body temperature, ElectroDermal Activity (EDA), motion and Heart Rate (HR) (Donaire-Gonzalez et al., 2019). In addition ‘Project Helix’ studied the environmental impact on individuals living in urban environments. Increased levels of blood pressure, asthma, allergy related illnesses and behaviour issues were found for those living in urban environments (Maitre et al., 2018).

Mobile technology in previous research coupled with sensors have aimed to provide a deeper understanding into the impact of exposure to an individual in a particular location. This highlights the potential of recent technological advances, whereby an individual’s exposure to the environment can be accurately assessed and calculated (Stamatelopoulou et al., 2018). Furthermore, particular areas have been found to have an increased risk of individuals developing serious health conditions such as higher heart rate, asthma and cardio-cerebrovascular disease (Kanjo et al., 2018). A study in 2018 used mobile technologies to develop the methods of assessing exposure to an individual. This involved using an activity and GPS sensor to predict an individual’s location. Overall the investigation demonstrated the capability of using sensors to accurately assess an individual’s exposure.

Personal sensors to measure individual exposure such as air pollution, noise, outdoor temperature, physical activity and blood pressure have been a positive way forward in monitoring due to their ability to collect data continually and in real-time helping to reveal early health conditions (Nieuwenhuijsen et al., 2014). By combining these sensor data streams together and the possibility for an individual to continuously wear sensors, the data can show the exposures an individual encounters as well as predict early health conditions (DeBord et al., 2016).

**Fig. 1:**
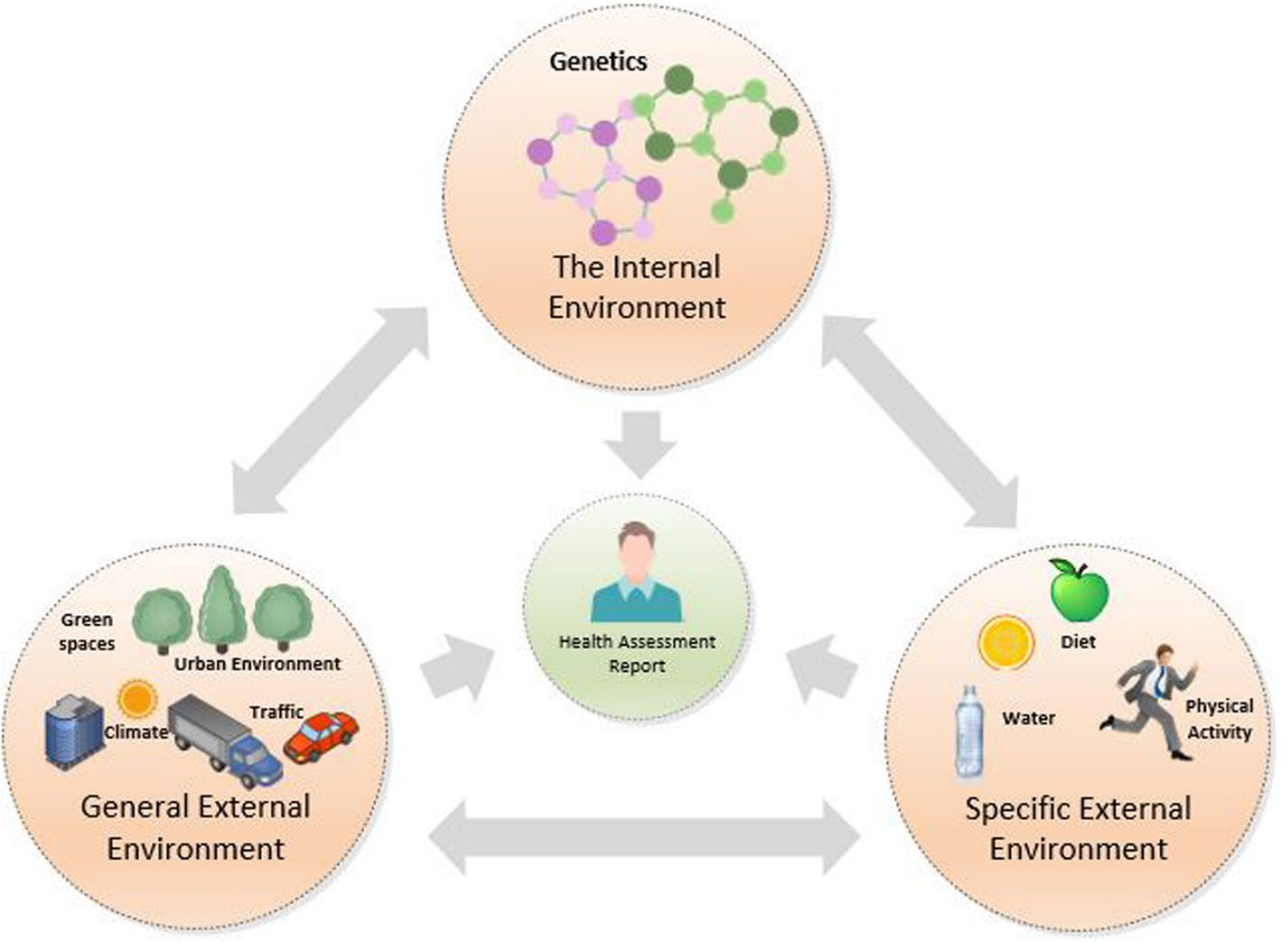
The three stages of the Exposome concept depicting how each plays a part in calculating the health assessment risk for an individual

Developed in 2005, the exposome concept encompasses each exposure that is subjected to a human from birth to death (Wild, 2012). In recent years, the concept is now actively being used in research communities as an alternative method to measuring the impact of the environment. Literature has already shown the impact of high polluted environments which have increased risk of developing conditions like asthma and cardio-cerebrovascular diseases (Loh et al., 2017; Maitre et al., 2018). Figure 1 presents the exposome concept in its simplest stage and highlights the large amount of data (e.g. Climate, Urban environment, Social, Diet, Physical Activity, Genetics) that is required in order to calculate exposure impact across an individual lifetime.

There are three stages associated with the exposome; internal, general exposome and specific external (Vrijheid, 2014). The first stage of calculating the exposome is, ‘internal’ that measures the body’s biological response to exposures; such as ageing and stress. The second stage, ‘general exposome’ considers the wider impact on our lives and influences on the individual such as their education background and financial situation. Finally, the ‘specific external’ which examines effects out-side of the body such as air pollution, radiation and diet. Several experimental studies on this concept have stated that once all three stages have been measured, the exposome can be exactly calculated (Wild, 2015).

## DigitalExposome

**Fig. 2:**
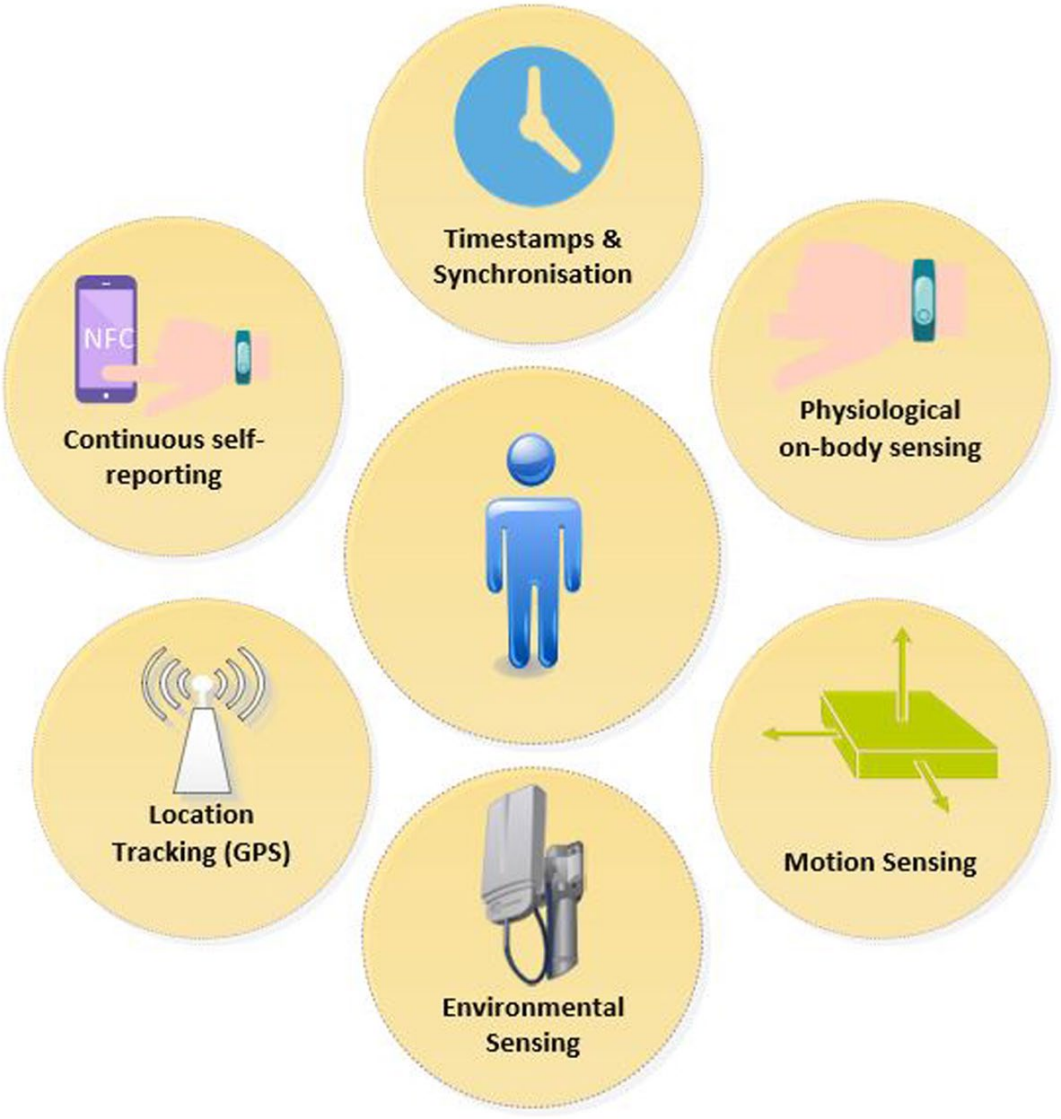
Data Collection methods to support DigitalExposome

We introduce the term ‘*DigitalExposome*’ as a framework to quantify an individual’s exposure to the environment by utilising a range of technological, mobile-sensing and digital devices, as shown in Fig. 2. This concept aims to measure multiple environmental factors using mobile technologies and then quantify them in real-life settings. Combining multiple data collection methods helps to support DigitalExposome and gain a better understanding into how exposures to the environment can impact mental wellbeing.

This concept, further promotes to the use of the exposome concept by digitally providing a better understanding into the impact of exposure directly to an individual. Through *DigitalExposome*, we aim to explore the opportunities that we for-see with this concept in exploring the link between pollution and wellbeing. To support this work, we have developed a range of sensing devices and applications. This involved a custom-build environmental monitoring system using a range off the shelf low-cost sensors, capable of sensing: Particulate Matter, Oxidising and Reducing Gases, Ammonia and Noise every 20 seconds with Internet-of-Things technology on-board. Readily available systems with these sensors built in are often very large, in fixed sensing stations and are not practical for ‘in-the-wild’ experiments.

*DigitalExposome* is primarily made up of two parts: data collection and data analysis. Both aspects make use of technological advances in order to calculate the exposome. In order to quantify the process, we propose the utilisation of data from sensors that show how an individual has been exposed to pollutants. We see this as being a key part of the exposome concept, where both terms are clearly connected through their vision of being able to capture the true exposure that an individual has been exposed to. Data generated through the use of technology, such as sensors offers new opportunities to link the exposure more directly to health.

## Methodology

### System architecture

**Fig. 3:**
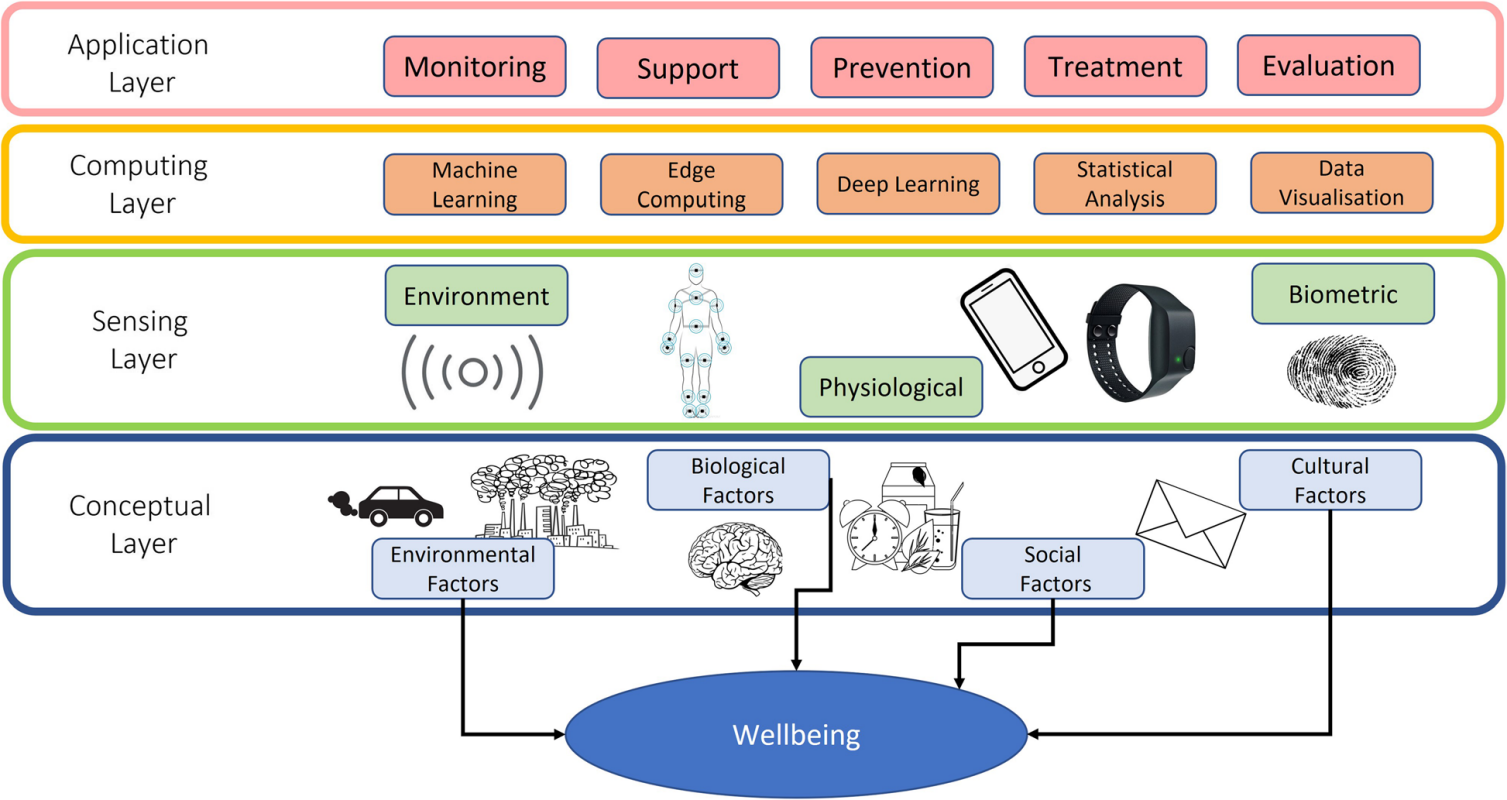
The conceptual and system architecture of DigitalExposome for calculating the impact to mental Wellbeing

Figure 3 presents the conceptual system architecture of DigitalExposome with four key layers. Firstly, the conceptual layer explains the four main areas that can impact mental wellbeing include environmental, biological, social and cultural factors (Liang et al., 2019). The sensing layer contains the physical devices (e.g. smartphone and wristband) and physiological systems to monitor HR, EDA and body temperature along with the environmental factors such as air quality. The computing layer lists several key core data science techniques that enables processing and analysis of the data including: Machine Learning, Deep Learning, Statistical Analysis and Data Visualisation. Finally, the application layer presents potential application scenarios of DigitalExposome for mental wellbeing. This can include the use of monitoring through the equipment described in Section 3; the use of green spaces in supporting prevention and treatment to result in a positive mental wellbeing.

### Experimental setup

**Fig. 4:**
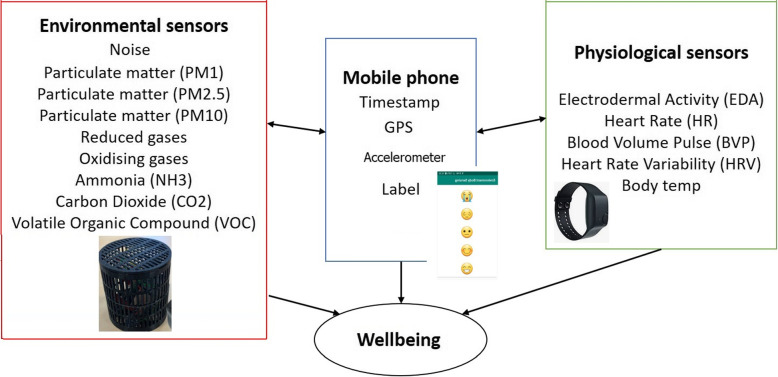
List of the fused variables collected by each device

Following ethical approval from Nottingham Trent University’s Ethics Committee, we recruited a total of 40 participants (25 Males and 15 females, aged between 18 and 50) who were all screened prior to the study, resulting in the total number of samples, after cleaning were 41,037. Previous literature has used a similar number of participants to carry out studies in the same area (Aspinall, 2015; Kinnunen et al., 2020). Participants’ were each provided with three devices; an environmental monitoring device (Enviro-Edge), Empatica E4 wristband and Samsung phone ready with the EnvoBodySends app. Each participant walked around a pre-specified route within an urban environment around Nottingham Trent University (Clifton Campus). Whilst walking, the three devices continually collected sensor data on environmental pollutants and physiological changes. In addition, participants self-reported their wellbeing continuously during the walk. The information acquired from each device is shown in Fig. 4.

The route was pre-specified, by selecting a mixture of urban environments from several green to busy and polluted spaces which would help to demonstrate the impact of different levels of exposure to air pollutants. Additionally, the journey taken by all participants took around 40 minutes to complete. This was decided due to previous user experience whereby they found it difficult to walk for longer. In addition, we did not want to exhaust participants which could have an impact on their body responses. Furthermore, studies with similar length of experiment time have found it difficult to motivate participants to walk further (Alajmi et al., 2013; Al-barrak et al., 2017; Kanjo et al., 2018).

**Fig. 5:**
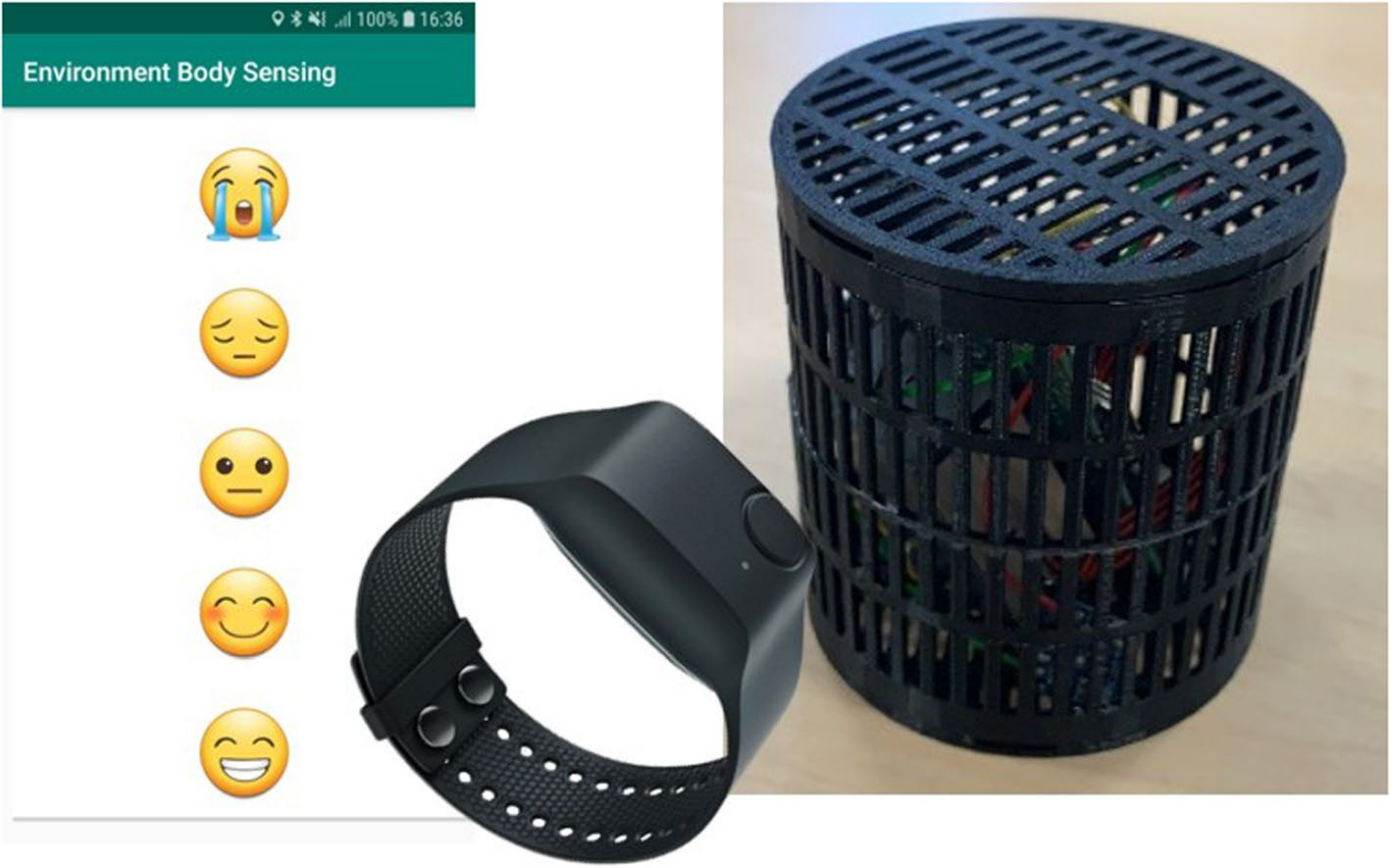
(left) Screenshot of smartphone application, (middle) E4 Empatica, (right) Environment monitoring kit

The experiment data collection tools are depicted in Fig. 5 that include the Enviro-IoT, E4 Empatica and smartphone application. The Enviro-IoT edge device equipped with a Raspberry Pi 4 records environmental data continually once every 20 seconds. While the E4 Empatica sensors’ data is sampled at different rates with HR at 1Hz and EDA, BVP, HRV and body temp at 64Hz.

Each participant used the custom built pre-installed “EnvBodySens” smartphone app to record their perceived wellbeing. We have adopted the ‘Personal Wellbeing Index for adults’ which asks the user how they are feeling with their life as a whole (Cummins & Ps, 2013). This has been adapted in the form of a five-point Likert SAM scale (Bradley & Lang, 1994) to provide a proven method for self-reporting subjective wellbeing. In our pre-installed mobile app the user is met with five well-know emojis, displayed on buttons from 1=negative/low to 5=positive/high . The idea is that the participant will be constantly prompted by the researcher to ascertain how they are feeling. Several studies such as Kanjo et al. (2018) and NeuroPlace (Al-barrak et al., 2017 have shown how momentary wellbeing labels can change quickly as moving through environments.

### Pre-processing

Following the data collection, the data was cleaned and pre-processed. Due to the varying sample rates, the physiological data collected (*EDA, BVP, HRV* and body temperature) were down-sampled to a rate of 1Hz to match the sample rate of collected HR by the device. In addition, the collected environmental sensor data had to be up-sampled to match the sampled rate of the physiological data at 1Hz. This was due to the low sample rate produced by the environmental device. Finally, the labelled data from the mobile smartphone was extracted and up-sampled to the same rate as the environmental and physiological data to 1Hz to remain consistent with the other data. To sample the data we have used linear interpolation (Needham, 1959). If the two known points are given by the coordinates (x_1_ , y_1_) and (x_2_ , y_2_). The linear interpolant is the straight line between these points. For a value x in the interval (x_2_ , x_1_), the value y along the straight line is given from the equation of slopes as shown below:

1 $$\begin{aligned} y=y_{1}+\left( x-x_{1}\right) \frac{\left( y_{2}-y_{1}\right) }{\left( x_{2}-x_{1}\right) } \end{aligned}$$

Following this, all signals were then normalised to bring all variables within the same range for both the data analysis and machine learning. Finally, all the sampled sensor data was fused together. Whilst cleaning the data, there were two variables excluded from the experiment; Carbon Dioxide and Volatile Organic Compound because of issues with logging the data resulting in no change in value during the experiment.

### Deep learning classification

To further explore the relationship of variables impact, machine learning and deep learning networks have been incorporated to classify the five self-reported states of wellbeing using the environmental pollution and physiological data from the 40 participants who successfully labelled their wellbeing. There were 3 participants whose data was removed prior to the classification due to issues around the self-recorded label and sampling of the environmental variables.

Deep learning presents many opportunities to extract features and classify raw sensor data. To enable the classification of the fused environmental, physiological and labelled data we employ a one dimensional Convolutional Neural Network (*CNN*) (Bai et al., 2018). Previous studies have shown how effective CNNs can infer mental wellbeing, particularly using physiological data (Woodward et al., 2020). Supervised CNNs are constructed by using numerous layers. These include the input, output and hidden layer which includes a convolutional layer that make use of a set of learnable filters, pooling layers, fully connected and normalisation layers (Yamashita et al., 2018).

In this work a one-dimensional CNN to classify the data, a CNN has also been used to extract features which were then used to train a number of machine learning classifiers. The extracted features from the CNN were used to train Random Forest, Support Vector Machine (*SVM*), Decision Tree, Gaussian Naive Bayes, Logistic Regression and Gradient Boosted supervised machine learning models to classify the five self-reported states of wellbeing using the pollution (*PM1, PM2.5, PM10, Oxidised, Reduced, NH3 and Noise*) and physiological (*BVP, EDA, HR, HRV* and body temperature) data. These machine learning models were selected due to their high popularity (Lisetti & Nasoz, 2004), trained over 20 epochs with a batch size of 128 and tested using 10-fold cross validation. The network architecture consists of 2 1-dimensional convolutional layers (64 and 32 neurons respectively) followed by a dropout layer with a rate of 0.5 to prevent over fitting before the ‘softmax’ activation function. Batch normalisation has been utilised within the network to normalise the inputs of each layer followed finally by a fully connection layer. The learning rate has been set at 0.001. Finally, the loss function used is Adam.

## Results

### Statistical factorial analysis and variable importance

We have employed mathematical and statistical approaches for the exploratory analysis stage including variable Correlations, *PCA* factor maps, variable importance and Pearson’s R Correlation Coefficient to measure the association between two categorical variables. Table 2 depicts a parameter value-based description showing values in this section.

**Table 2:** Parameter Value-Based Description of Sensor Units

Parameter	Value Description
dB	Decibels
ms	milliseconds
$\upmu$g/m3	one-millionth of a gram per cubic meter air
$\upmu$S	Microsiemens
ppm	parts per million

**Fig. 6:**
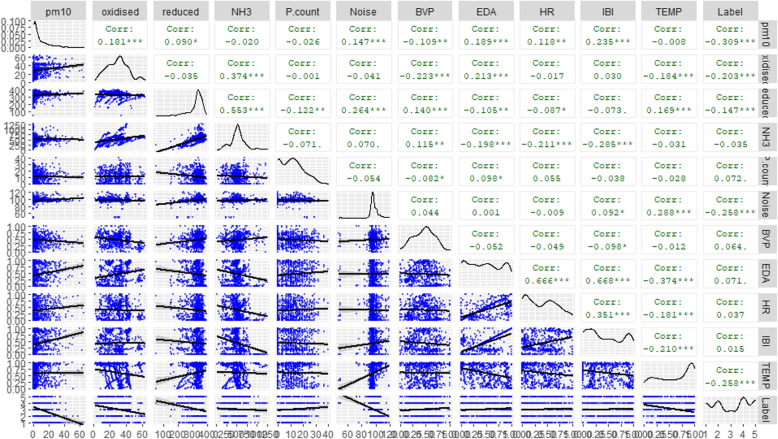
Correlation Matrix of the Environmental and Physiological Variables

A correlation matrix has been depicted at Fig. 6 to further understand the relationship between the different variables. From the matrix, it is clear to see that some variables are highly correlated together. Analysing the individual cells shows HRV correlates well with PM10 and NH3. In addition, EDA significantly correlates with PM10, Oxidised and Reduced gases and NH3.

*PCA* Factor Maps are an effective method for large datasets, to help understand the relational impact between different variables, with reducing information loss (Jollife & Cadima, 2016). Also, using PCA maps provides a visual method of presenting data and observing correlations between different variables (Kanjo et al., 2018). PCA factor maps give a view of all the variables projected on to a plane, spanned by the first two principle components. This method demonstrates the structural relationship between the different variables. We have demonstrated two PCA factor map plots based on variable importance of the many variables collected.

**Fig. 7:**
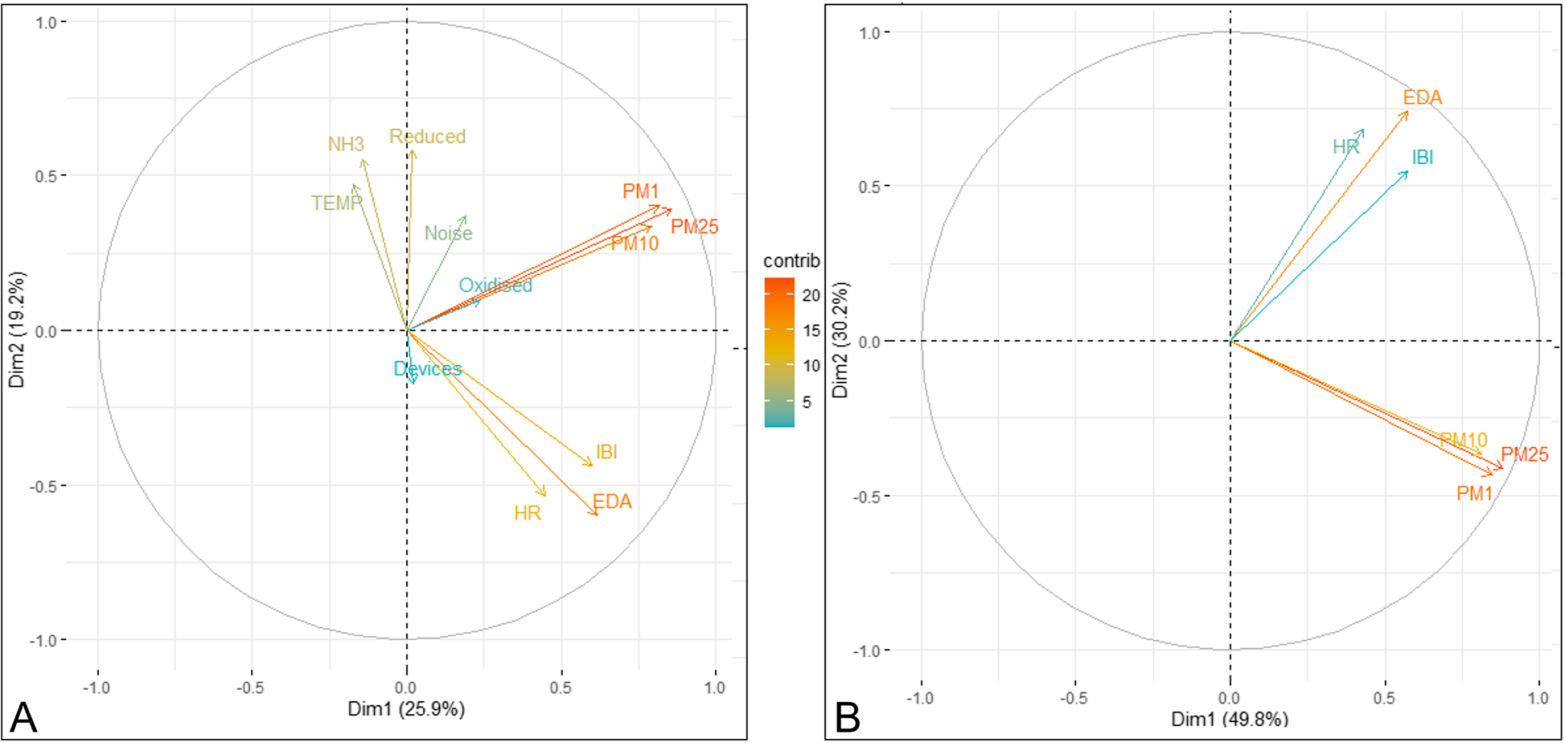
PCA Analysis - **A** Variance between the different variables, **B** Variance between the different variables without EDA

Figure 7 (A, B), presents the captured environmental and physiological variables depicted on a PCA map. It is worth noting, that most of the body attributes EDA, HR and HRV are all at the top of the figure, while, the environmental variables PM1, PM2.5, PM10 and Reducing gases are located in the middle. From the diagram (A), there is Dim1 25.9% and Dim2 19.2%, resulting in 45.1% in total variance across the environmental and physiological variables. It is worth noting that the most important, (or, contributing) variables are highlighted using the colour gradient (i.e. darker colours indicate higher contributing factor).

At the second PCA diagram (B), depicts the most important variables as identified at diagram (A) including PM1,PM10, PM2.5, HR and IBI with the least contribution variables discounted. With this PCA diagram we notice that the total variance increases to 80% made up from Dim1 49.8 and Dim2 30.2%. Higher increased of variance in other studies has shown the stronger association between variables (Rosenthal, 2012), as evident in PCA diagram (B). The close grouping and proximity of the independent variables suggests that HRV, HR and PM10 are correlated and that HRV, HR, PM2.5 can also be correlated. Analysing these early findings indicates that lower the HRV and higher HR is correlated to a higher level of air pollution within the environment.

**Fig. 8:**
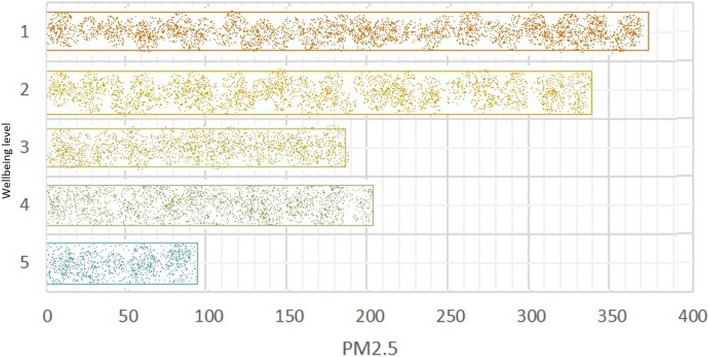
Depicts the relationship between the self-reported Participant’s wellbeing (Label) and PM2.5

Furthermore, Fig. 8 demonstrates the impact of wellbeing against levels of PM2.5 within the environment. The bars on the chart are associated with how many times a particular user would label how they were feeling (reported wellbeing) whilst walking around the environment. The results of this indicate that high levels of PM2.5 are associated with a negative wellbeing, shown by participants choosing ‘1’ on the device. Whereas where participants labelled ‘5’ (very positive wellbeing), the levels of PM2.5 were much lower. This early analysis on the collected sensor data helps to understand the impact of pollution on mental wellbeing.

### Multi-variant regression analysis

Using a PCA analysis and covariance matrix enables the exploration of the relationship between the obtained variables. We continue this process by using multi-variant regression to understand the importance each variable has on the other. In the case of this work, we explore each of the dependent variables (physiological data) to compare against the independent variables (environmental data).

**Multiple Regression Model for EDA:** Firstly, a multiple linear regression module for EDA has been used to understand the impact of this physiological on-body sensor to the other independent environmental variables including NH3, Noise, PM1, PM2.5, PM10 and Reduced. Table 3, shows the multiple regression results for EDA.

**Table 3:** Multiple Regression Analysis between EDA and Environmental variables

	Coefficients	Standard Error	t Stat	*P*-value
Intercept	-0.02381894	0.02225209	-1.07041	0.284452
nh3	0.000291595	$1.14608 \textrm{E}-05$	25.44285	$1.5 \textrm{E}-139$
noise	0.004050864	0.000221511	18.2874	$7.92 \textrm{E}-74$
oxidised	-0.00590754	0.000143065	-41.2928	0
pm1	-0.00768185	0.00081832	-9.38735	$7.11 \textrm{E}-21$
pm10	0.000939923	0.000285371	3.29369	0.000991
pm25	0.003698711	0.000800215	4.622149	$3.83 \textrm{E}-06$
reduced	-0.00058528	$4.59985 \textrm{E}-05$	-12.7239	$7.06 \textrm{E}-37$

**Fig. 9:**
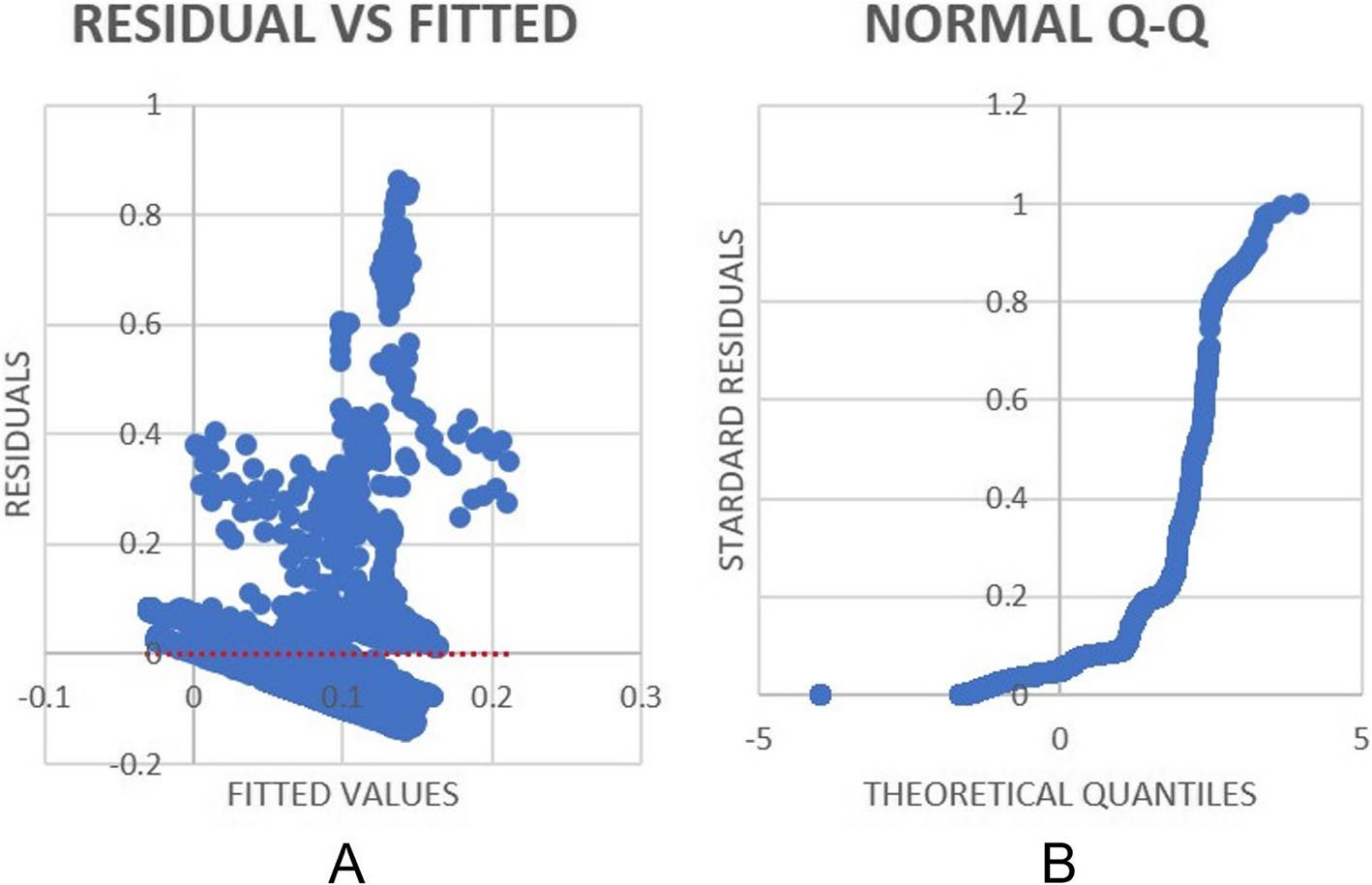
EDA Regression: Residuals VS Fitted values curve (**A**) and Q-Q Plot (**B**)

At Table 3, the coefficients demonstrate that the environmental variables (NH3, Noise, PM10 and PM2.5) involves an increase in EDA. A negative coefficient shows that as EDA increases the remaining environmental variables decrease showing that there is a less of association between them. In addition, a negative (-) t-stat value for each environmental variable depicts a negative impact on the variable of EDA. Whereas a positive value indicates an association between the environmental variable and EDA. The data in Table 3 was then evaluated using a regression curve shown in Fig. 9. This shows the relationship between the calculated residual values verses the fitted values shown at (A) and (B) respectively.

Figure 9 depicts the graphs of Residuals VS Fitted (A) and a normal Q-Q plot (B) for EDA by using bi-modal data. The aim of a Residual VS Fitted graph is to ascertain whether linearity holds which is normally indicated by the mean of the residual values being close to 0. In the case of (A), this is shown by the red dotted line being close to 0. On the other hand, the Q-Q plot (B) is used to in order to fit a linear regression model. In many Q-Q plots, the data on the graph takes the shape of a twist like seen in this plot (Kanjo et al., 2018; Scott, 2015). This plot is presenting a symmetric distribution with ‘fat-tails’, otherwise known where the ends of the line curve. The lower part of the plot is almost linear, suggesting a normal distribution in relation to one mode of data distribution. In addition, the upper part of the Q-Q plot again suggests linear, showing an approximate distribution. The steep line between the upper and lower curve is steeper than the line y = x which suggests the distribution plotted on the vertical axis is more dispersed than the distribution plotted on the horizontal axis. The implication to this is that the data points are normally distributed.

**Multiple Regression Model for HR** Below presents the multiple linear regression model for HR using the other independent variables (environmental). This includes: NH3, Noise, PM1, PM2.5, PM10 and Reduced. Table 4, shows the multiple regression results for HR:

**Table 4:** Multiple Regression Analysis between HR and Environmental variables

	Coefficients	Standard Error	t Stat	*P*-value
Intercept	128.842080	1.7214547	74.844883	0
nh3	0.00732292	0.0008866	8.259339	$1.598 \textrm{E}-16$
noise	-0.08328681	0.0171364	-4.8602191	$1.185 \textrm{E}-06$
Oxidised	-0.05183384	0.0110676	-4.6833448	$2.849 \textrm{E}-06$
pm1	0.11853817	0.0633064	1.8724500	0.06116
pm10	0.11218463	0.0220767	5.0815832	$3.792 \textrm{E}-07$
pm25	-0.23280472	0.0619057	-3.7606292	0.000170
reduced	-0.07204261	0.0035585	-20.245134	$8.125 \textrm{E}-90$

The findings in Table 3 are in agreement with previous research that shows as PM1.0 and PM10 increase result in an increase in Heart Rate due to the highest positive coefficient reading (Rumchev, 2018). In addition, NH3 shows a very small positive coefficient showing impact towards HR. Finally, research has shown how differing levels of irregular environmental noise can impact a regular heart-beat. In particular, recent studies exploring this find that noise levels between 55 and 75 Decibels (dB) are linked to a higher risk of developing heart related diseases (Münzel et al., 2014).

**Fig. 10:**
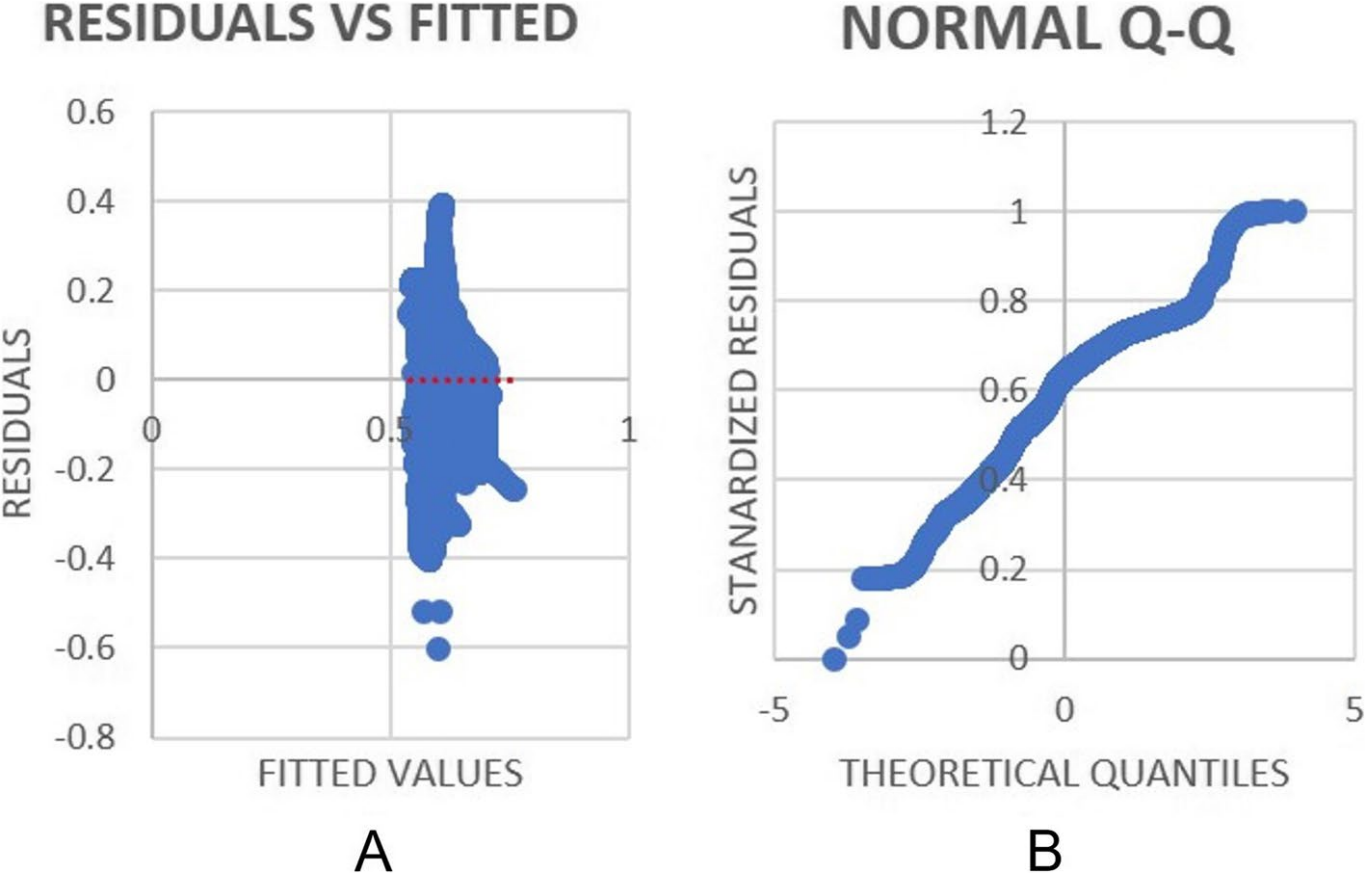
HR Regression: Residuals VS Fitted values curve (**A**) and Q-Q Plot (**B**)

Figure 10 depicts the Residual VS fitted values and normal Q-Q Plot as shown at A and B respectively. Similar to the EDA Q-Q plot, the HR Q-Q plot demonstrates a twist at either end of the plot. In addition the data shows a clear bi-modal distribution. The lower part of the plot is almost linear suggesting an approximate normal distribution. The line in the middle of the upper and lower parts follows a more linear (y=x) line, meaning that the distribution is less dispersed. It is worth noting that there were three outliers for HR distribution due to erroneous sensor readings.

### Spatial visualisations

**Fig. 11:**
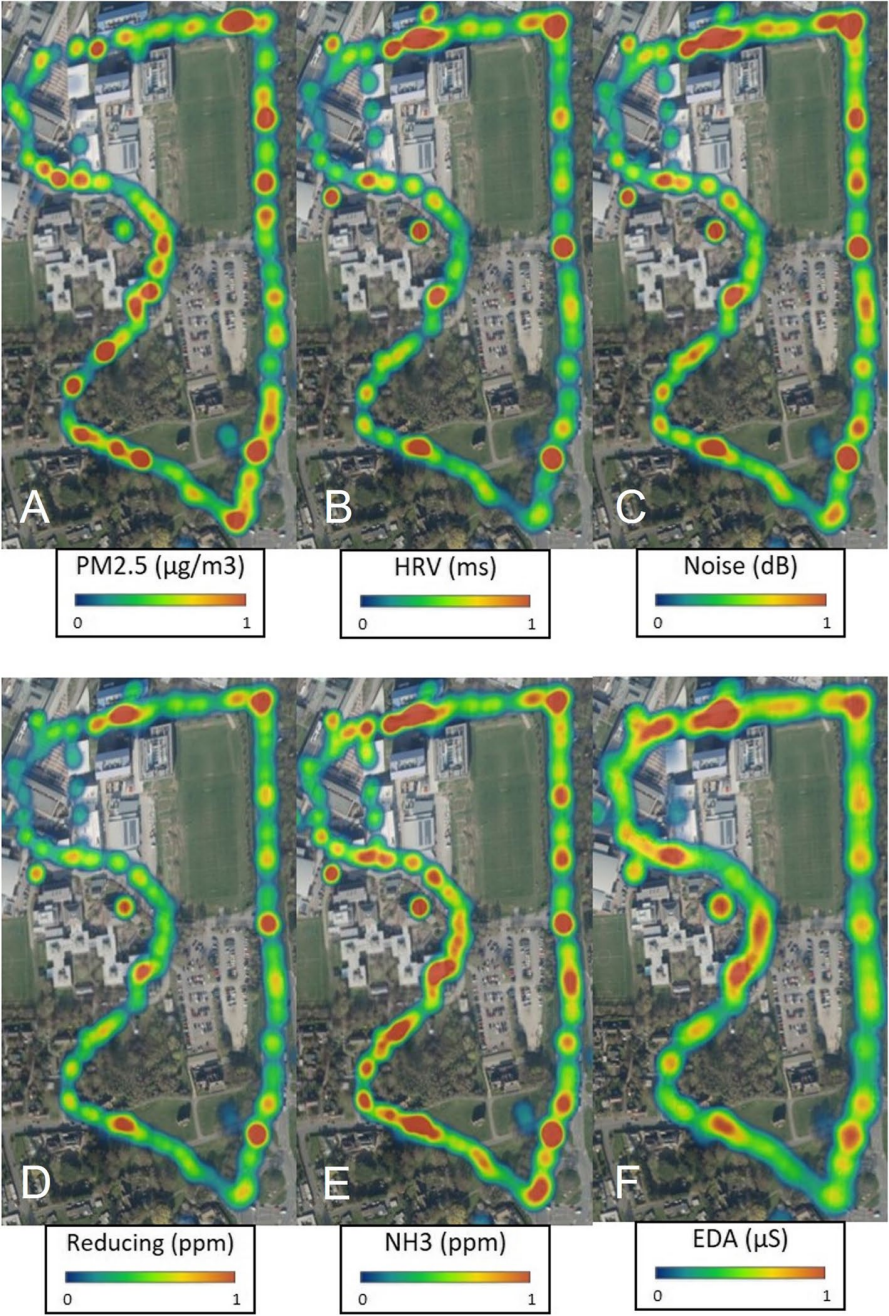
Several heatmaps (**A**, **B**, **C**, **D**, **E**, **F**) indicating the changing of environmental and physiological sensor data as participants move between environments

To summarise the dynamic sensing patterns and act upon the findings using visualisation, the geographical study area needs to be divided in smaller areas. One common way of looking at patterns is to use heat maps to visualise the sensor data. Mapping of sensor data in this way has in other studies been a proven method in visualising dynamic data (Mashima, 2012). For example, Fig. 11 presents six heat maps plotting environmental and physiological sensor data, showing the changes whilst the participant travelling along the route. In particular, observations on the (upper right of the maps) show that each participant was subjected to an increase of PM2.5 and Noise and was met with an increase of HRV and EDA. This approach further demonstrates the impact of the environment on mental wellbeing states.

Figure 11, depicts the sensor data hot-spots which are scattered along the path with varying levels of intensity. Results from this visualisation further demonstrate that when participants are met with high levels of pollution, particularly PM2.5 (A), Noise (C), Reducing (D) and EDA (F), directly impacts HRV (B) and EDA (F). While the heat maps show the level of intensity based on GPS traces coordinates, the sensor data on these heat maps indicate the real distribution of sensor data. One option is to divide the study area into grid cells (Kanjo, 2010) however, it is difficult to allocate a cell to each sensor reading, moreover, it is not possible to decide on the cell size, since the density of the sensor mobility traces can be of different density distribution.

To address these issues, we utilise combinatorial computational geometry algorithm called “Voronoi”, which is a diagram partitioning of a plane into regions based on distance to points in a specific subset of the plane (Dobrin, 2015). The method of Voronoi visualisations is a computational geometry algorithm which allows the visualisation of large data sets (Dobrin, 2015). The concept works by defining a set of polygon regions called cells, whereby the cells give an indication of the overall density of an object area of the size of the object itself (Pokojski & Pokojska, 2018).

Voronoi Diagram divides the space into a set of regions called Voronoi cells, including the space that is closest to the object (route location, in our case). The size of these cells gives an indication of the density of the area a certain object is in or the size of an object (Pokojski & Pokojska, 2018). The cell structure also shows the Delaunay triangulation, which easily allows calculating an object’s immediate set of neighbours. The definition of a Voronoi cell is given by the following equation, where x is a planar metric space; p is the set of generator points in the metric space; and d is the distance between all points in x and a specific generator point (where the distance can be defined using any distance definition such as Euclidean, Manhattan, or road-network distance):

2 $$\begin{aligned} Vor{_i}=\left\{ x \mid d(x,p{_i})\le d(x,p{_j}),j\ne i \}\right. \end{aligned}$$

Thus, the Voronoi diagram is composed of a collection of tessellations (i.e. polygons) defined as Vor, where:

3 $$\begin{aligned} Vor{_i}=\left\{ Vor{_1}, Vor{_2}... Vor{_n}\}\right. \end{aligned}$$

The creation of a Voronoi tessellations is a dynamic procedure till all the points are represented in adjacent polygons. If sufficient number of particles did not satisfy Equation (1) then Voronoi gets partially filled. In this case, the data is then redistributed. By giving each polygon a class value Ci that corresponds to the sensor value collected in a particular GPS coordinate, it is then possible to divide the space into adjacent polygons with different sensor reading which are represented in colours.

**Fig. 12:**
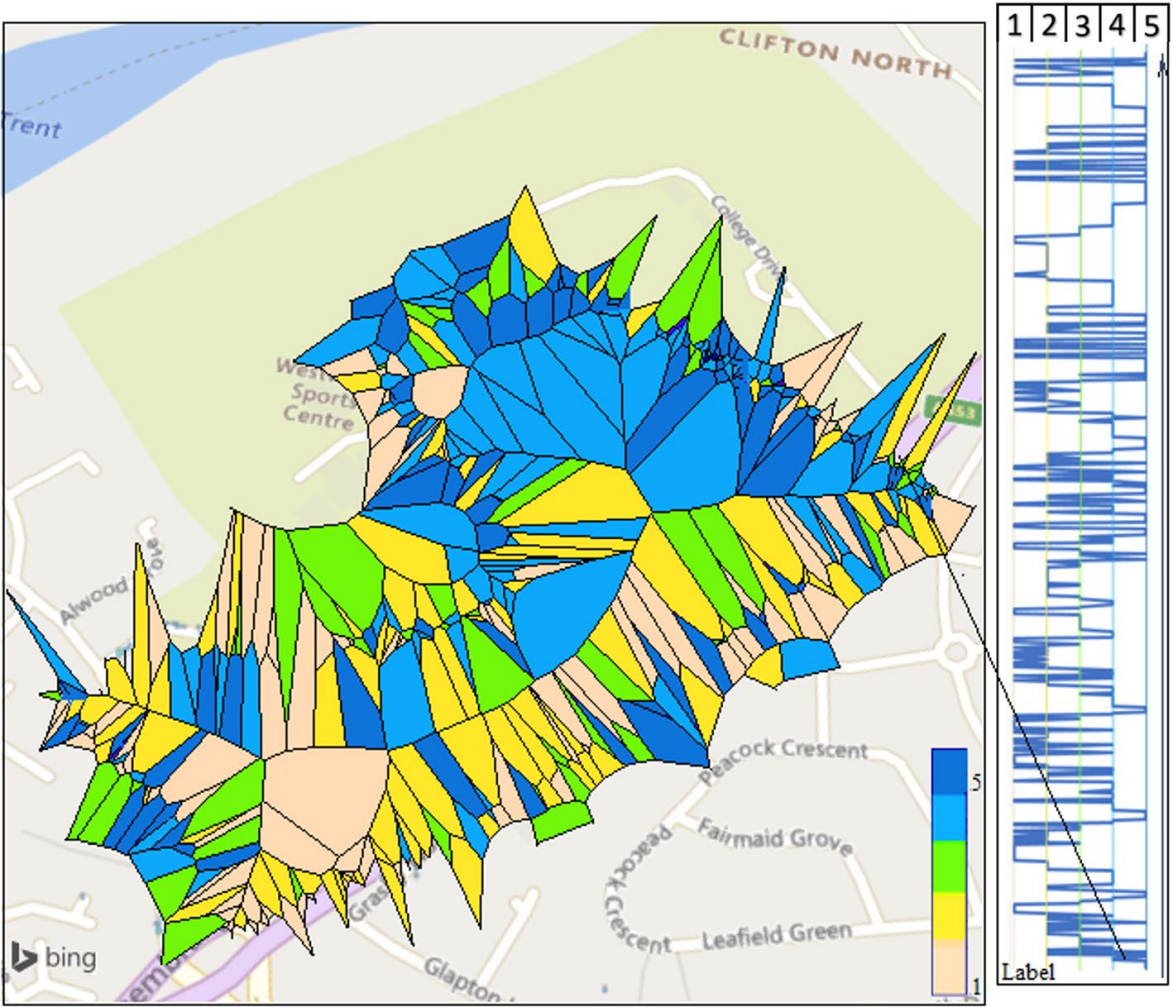
(left) Voronoi overlay from one participant data. Each polygon represents one location trace tagged with a wellbeing label while collecting the data in specified route (the map layer from Microsoft Bing), (right collected label data from start to end)

Figure 12, presents the self-reported wellbeing data using the app on the specified route for this experiment. The color of the polygons represents the wellbeing data from low negative to high positive. The visualisation demonstrates that poor wellbeing (lighter colour; i.e. cream and yellow) was most reported along the main road where high levels of pollution were also experienced whereas more positive states of wellbeing was recorded in less polluted areas such as fields and open spaces (dark colours; i.e. blue ). On the right of the Voronoi, demonstrates the labelled data from a participant while walking along the route. As an example, the arrow shows that when participants are met with a change in the environment, they label as being unhappy.

### Classification results

**Fig. 13:**
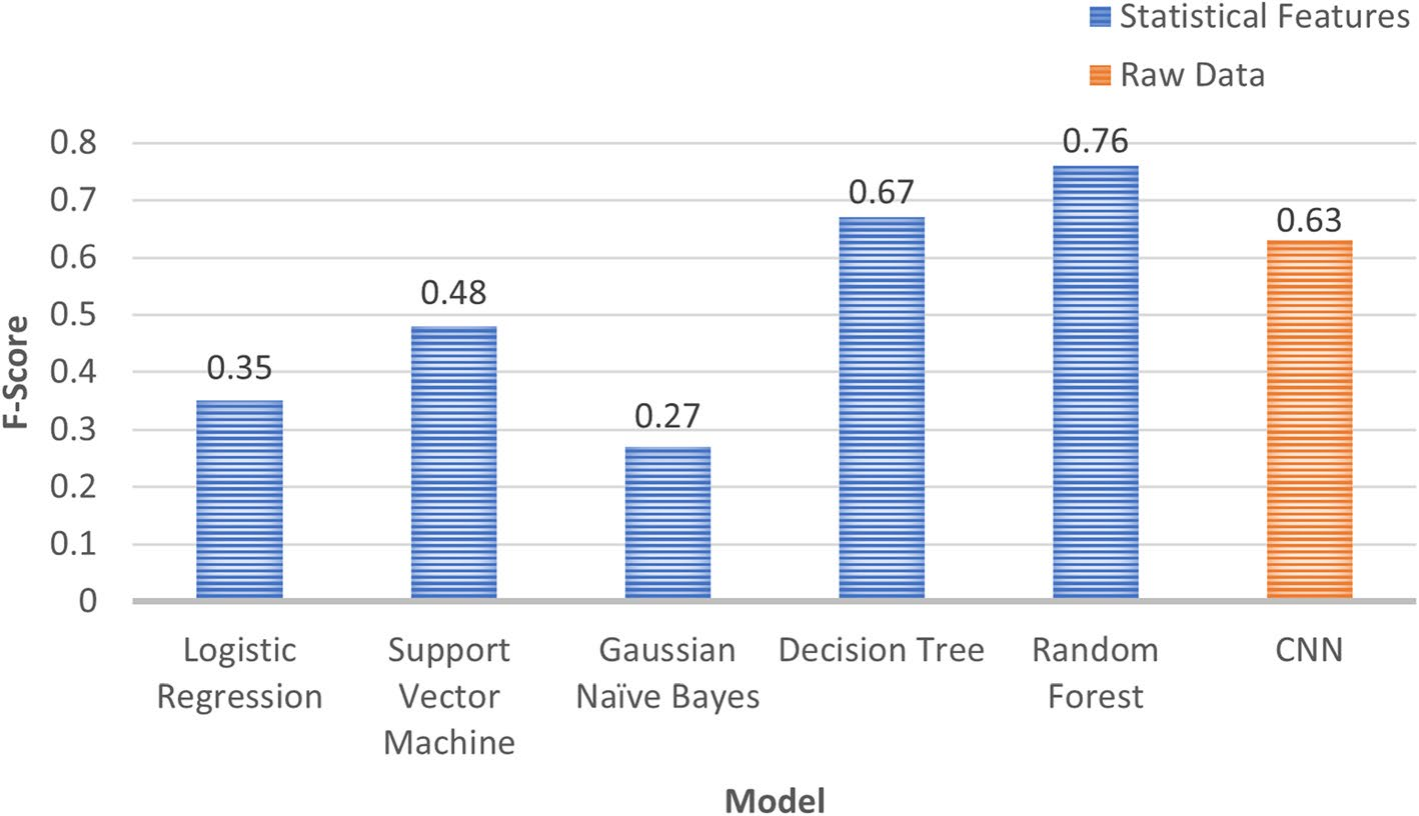
Comparison of classification models trained using statistical features and raw data from a CNN

**Fig. 14:**
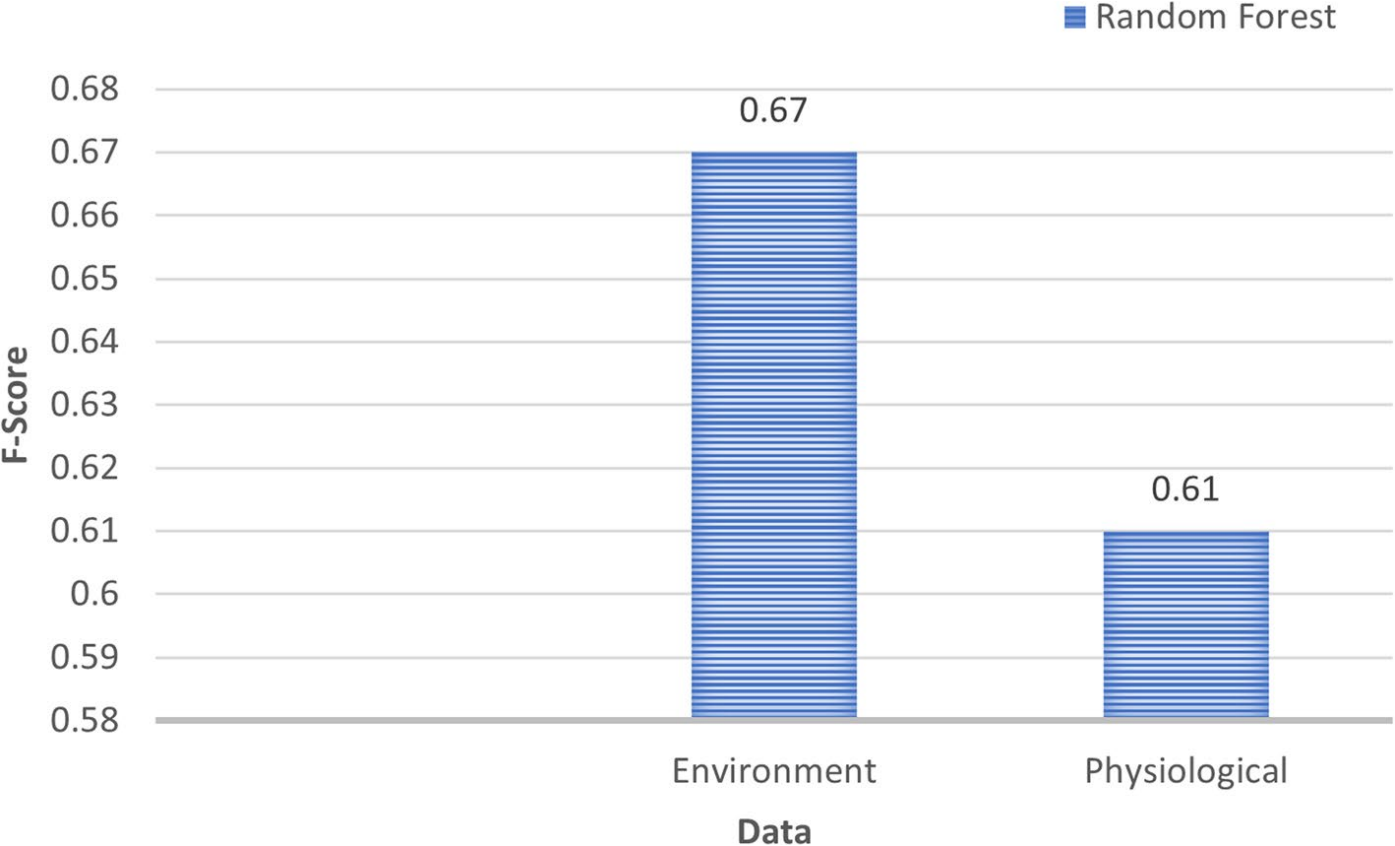
Comparison of Random Forest combined with CNN when trained using only the environmental or physiological data

Figure 13 presents the f-score for each of the classification models trained using standard statistical features. The Random Forest classifier was the best performing model achieving an f-score of 0.76, outperforming the other statistical models by 0.09 and the CNN which is frequently used for wellbeing classification by 0.13. To further explore the environmental impact on mental wellbeing, the best performing classifier (Random Forest) was trained using the environmental and physiological data separately which achieved an f-score of 0.67 and 0.61 respectively, as shown in Fig. 14.

Analysing the results further in terms of precision and recall across the six different models for classification the scores were very similar in values. In particular, Logistic Regression and Support Vector Machine scored very low on precision and recall (0.44 and 0.33 respectively), struggling at predicting the middle of labels (3 and 4). At the two highest achieving models (Decision Trees and Random Forest) both were similar resulting in higher precision and recall values (0.70 and 0.74 respectively), with slightly lower scores of 0.33 when predicting label 2.

The results from Fig. 14 indicates that wellbeing can be inferred using environmental data alone, achieving an f-score of 0.67 while wellbeing can be inferred from the physiological data with an f-score 0.61. As previous studies have shown, it is expected that the physiological data would accurately classify wellbeing due to its high correlation with the sympathetic nervous system (Sharma & Gedeon, 2012). However, it is interesting that pollution data combined with physiological data outperformed the model trained using pollution data alone, demonstrating the benefits of pollution data. Furthermore, the CNN trained using only pollution data outperformed the CNN trained using physiological data, suggesting pollutants have a considerable impact on wellbeing.

## Discussion and limitations

Continually collecting and fusing real-world environmental and physiological sensor data helped us learn about our surroundings and how we interact and behave in different environmental conditions. This has gone beyond previous work in this area which typically only observes how noise can impact wellbeing (Kanjo, 2010) and does not consider other environmental pollutants. A PCA analysis suggests that when all collected variables are combined together they can describe the variability of the data as a whole. In particular, on the PCA map, the physiological sensors (*EDA, HR and HRV*) point towards a different location to the environmental variables. From our analysis we can conclude that a range of environmental factors PM1.0, PM2.5, PM10 impact physiological changes HRV, HR. The Multi-Variant Regression analysis has further unravelled the relationship the environment and physiological variables, in particular focused on HR and EDA.

Voronoi visualisations have given an indication of how changes within the environment can have an impact on mental wellbeing. Typically, it was found that where air pollution such as PM1, 2.5, 10 and noise was increasing, participants labelled their wellbeing as very negative. This demonstrates consistent results with previous studies in this area (Kanjo, 2010; Johnson et al., 2020). This form of spatial analysis, greatly helps in understanding the degree to which a place is similar to other nearby places.

The ability to classify the collected data presents many possibilities for the real-world inference of wellbeing using pollution data. The results show that using features extracted from a CNN successfully improved the accuracy in which wellbeing can be inferred. Combining physiological with environmental pollution data achieved an f-score of 0.76 compared with an f-score of 0.61 when trained using only physiological and 0.67 when trained using only environmental pollution data. The ability for pollution data to increase overall f-score demonstrates its impact on wellbeing and shows pollution should continue to be considered as a factor that influences changes in wellbeing. Furthermore, the best performing model was a Random Forest trained using features extracted from a CNN. The ability for machine learning models such as the Random Forest Classifier to outperform a CNN demonstrates the benefits of using a CNN to extract features and train using a separate classifier.

During this study some limitations were encountered. Early analysis on the collected sensor data found that the Empatica E4 was not reliably collecting participants’ EDA. While the EDA sensor worked successfully for some, for other participants no variation in EDA was recorded throughout the experiment. At the point of fusing the collected sensor data, both CO2 and VOC were found to have collected data for some participants but not all, resulting in its dismissal. As this study was conducted during the COVID-19 pandemic, collecting data in a participant heavy experiment was challenging task. In the future, we aim to recruit more participants to further investigate and generalise the relational impact of the environment on mental wellbeing.

## Conclusion

In this paper, we proposed the new concept ‘*DigitalExposome*’ that demonstrated the potential of employing a multi-model mobile sensing approach to further understand the relationship between the environment and its impact on mental wellbeing. To achieve this, a real-world experiment was conducted where participants walked around a specified route reporting their responses and collecting environmental, behavioural and on-body sensor data. Several statistical analysis techniques were used including PCA, Multi variant Linear Regression, Voronoi and data spatial visualisations were implemented to explore the variation in data and the factor importance. In this study, we found that physiological (on-body) sensor data is directly correlated with high levels of pollution (particulate matter in particular) within the environment. In addition, CNNs have helped successfully classify five states of wellbeing achieving an f-score of 0.76 using the fused physiological and pollution data. Sensing technology can shed the light on how people breath, feel and interact with their environment in different surroundings. This can help in offering a better security for city dwellers and creating a bond with their environments. In the future, we hope to consider additional environmental sensors to observe greater changes that may improve our sense of places and characterize the relationship between people and spatial settings, which in turns might influence the future design of urban spaces.

## Data Availability

There are no plans to make the data available at the moment due to the guidelines given by Nottingham Trent University’s Ethics Committee.
